# Multigrain Bread: Impact of Germinated Grain Supplement on Phytochemical Profile and Technological and Nutritional Properties

**DOI:** 10.3390/foods15061029

**Published:** 2026-03-16

**Authors:** Andrej Živković, Tomaž Polak, Tomaž Požrl

**Affiliations:** Department of Food Science and Technology, Biotechnical Faculty, University of Ljubljana, SI-1111 Ljubljana, Slovenia; andrej.zivkovic@bf.uni-lj.si (A.Ž.); tomaz.polak@bf.uni-lj.si (T.P.)

**Keywords:** germinated grains, multigrain bread, secondary metabolites, LC–MS/MS, phenolic compounds, antioxidant activity, dietary fibre, technological properties

## Abstract

Modern diets often provide insufficient health-promoting nutrients, prompting the development of enriched staple foods. This study investigated the impact of incorporating germinated spelt (*Triticum spelta*), naked oat (*Avena nuda*), and buckwheat (*Fagopyrum esculentum*) seeds at 30% and 60% levels on the nutritional, technological, and sensory properties of wheat bread. Liquid chromatography–mass spectrometry (LC–MS/MS) analysis verified the successful transfer of grain-specific bioactive compounds into the dough and bread matrix—benzoxazinoids (BOA, MBOA) from spelt, avenanthramides (AVN A, B, C) from oats, and flavonoids (e.g., rutin, vitexin, orientin) from buckwheat—emphasizing both free and bound metabolite fractions. Multigrain breads exhibited a complementary phytochemical profile. The antioxidant properties of the enriched breads were markedly enhanced, with germinated buckwheat providing the most pronounced increase. Analysis confirmed a significant increase in dietary fibre content proportional to the level of germinated grain addition, with almost double the content in 60% multigrain bread. Texture analysis indicated that the control crumb exhibited the greatest relative firming over 48 h during storage. Sensory evaluation showed that all of the enriched breads received high acceptability scores (>18/20). The incorporation of germinated seeds effectively enhances the nutritional value of bread, offering a promising strategy for developing health-promoting bakery products.

## 1. Introduction

The modern way of life is often associated with unbalanced dietary habits that provide insufficient nutritionally important ingredients such as key vitamins, antioxidants, minerals, and fibre [1]. The resulting deficiencies can lead to various health issues and chronic conditions with impacts on overall health. Enriching staple foods with nutrient-rich supplements offers numerous benefits, such as improving public health and reducing nutritional deficiencies. It is a cost-effective method for delivering nutrients to a large population. Cereals and their products are widely consumed, making enrichment a practical method for reaching various populations, without significant changes in dietary habits.

Germination is a natural process that improves the bioavailability of nutrients, making edible seeds an excellent functional ingredient in the baking industry. The enrichment of bread with germinated seeds provides numerous functional properties. Germination increases the levels of vitamins and various secondary metabolites, and antioxidant properties [2,3,4]. Higher levels of different plant secondary metabolites such as polyphenols and flavonoids can help to protect cells from oxidative stress, reducing the risks of chronic diseases and certain cancers [5]. In addition to enhancing the accumulation of secondary metabolites, germination induces a wide range of biochemical and structural modifications in seeds. During this process, hydrolytic enzymes such as amylases, proteases, and phytases are activated, leading to the partial degradation of starch and storage proteins, as well as reductions in antinutritional factors such as phytic acid. Germination may improve mineral bioavailability, protein digestibility, and overall nutritional quality. The addition of germinated wholegrain seeds in wheat bread can improve the texture, flavour, antioxidants, and dietary fibre content [6,7,8]. This makes enriching bread with germinated seeds an excellent choice for consumers seeking healthier, more nutrient-dense bread options.

In this study, we investigated the effect of adding germinated spelt (*Triticum spelta*), naked oat (*Avena nuda*), and buckwheat (*Fagopyrum esculentum*) seeds on the nutritional, technological, and sensory properties of prepared wheat breads. These species were selected due to their distinct and complementary phytochemical profiles. Germinated spelt is characterized by the accumulation of benzoxazinoids [2], cereal-specific secondary metabolites that are increasingly recognized for their potential biological activity. Naked oat is unique for its content of avenanthramides—phenolic alkaloids found almost exclusively in oats and known for their strong antioxidant [4] and anti-inflammatory properties. Buckwheat, a pseudocereal, differs substantially from true cereals in its flavonoid-rich phenolic profile, being especially rich in rutin, orientin, isoorientin, and vitexin, which increase during germination and contribute to higher antioxidant activity [3]. The differences in the most important classes of secondary metabolites among these three species are the main reason for their combined use. This diversity may broaden the spectrum of bioactive compounds and potentially enhance the functional value of the final product.

An important issue regarding the bioavailability of secondary metabolites in bakery products is their thermostability during baking. The effects of thermal treatment depend on the phenolic species and treatment conditions, meaning that either an increase or decrease in phenolic content may occur. Thermal processing may, for example, increase the content by breaking down cell walls in cereal products or decrease it as a consequence of thermal degradation [9]. Secondary metabolites in our experiment, such as flavonoids and avenanthramides, are generally vulnerable to thermal processing [10,11]. To the best of our knowledge, there are no published reports on the thermal stability of benzoxazinoids; therefore, our comparison of data before and after baking represents a small contribution to an important area of research that remains relatively unexplored. In addition to beneficial nutritional and health effects, enrichment with germinated seeds can provide additional benefits such as improved sensory and rheological properties.

In cereals, phenolic compounds occur in both free and bound forms, and when evaluating their overall contribution in cereal products, it is important to consider both fractions. Free phenolics are readily extractable and more frequently reported in the literature, but they represent only a minor proportion of the total phenolics in grains. The bound fraction is often neglected, despite the fact that it represents a substantial proportion of total phenolics [12]. Bound phenolics are important because they can resist digestion in the upper gastrointestinal tract and reach the colon, where they can contribute to a favourable intestinal environment [13]. Therefore, assessing both free and bound fractions is essential for an accurate estimation of the phytochemical profile and potential functional value of cereal-based products.

The present study was designed to systematically evaluate how the incorporation of germinated spelt, naked oat, and buckwheat influences the phytochemical profile and overall quality of white wheat bread. Particular attention was given to the transfer of grain-specific secondary metabolites—benzoxazinoids, avenanthramides, and flavonoids—in free and bound fractions using LC–MS/MS analysis. In addition, changes in total phenolic content, antioxidant activity, dietary fibre composition, and key technological and sensory parameters were evaluated to determine whether the high-level incorporation of germinated grains can produce nutritionally important improvements while maintaining acceptable bread quality.

## 2. Materials and Methods

### 2.1. Materials

In this study, we used the ‘Čebelica’ cultivar of buckwheat, the ‘Kamil’ cultivar of naked oat, and the ‘Ostro’ cultivar of spelt, all organically produced and harvested in the year 2023 by a local producer (Krasinec, Slovenia). The grains were stored in paper bags under dry and dark conditions at 4 °C until use. The selected cultivars represent commercially important varieties for cereal production in Slovenia. Sodium hydroxide, methanol, 1,1-diphenyl-2-picrylhydrazyl (DPPH reagent), trolox, and sodium bicarbonate were obtained from Sigma-Aldrich (Sigma-Aldrich GmbH. Steinheim, Germany). Folin–Ciocalteu (FC) reagent and formic acid were obtained from Merck (Darmstadt, Germany), and manganese dioxide was obtained from Kemika (Zagreb, Croatia). The analytical standards of rutin (PN: 78095-25MG-F), orientin (PN: 09765-1MG), isoorientin (PN: 78109-5MG), 6-methoxy-2-benzoxazolinone (MBOA), 2-benzoxazolinone (BOA), catechin (PN:43412-10MG), epicatechin (PN: 68097-10MG), *p*-coumaric acid (PN: C9008-10G), ferulic acid (PN: 128708-25G), vitexin (PN: 49513-10MG-F), avenanthramide A (PN: 30366-10MG), avenanthramide B (PN: 93105-10MG), avenanthramide C (PN: 36465-10MG), and cis/trans-isomers of ferulic acid (PN: 9004-100MG) were purchased from Sigma-Aldrich (Steinheim, Germany). Schaftoside (LOT: 739322001) and isoschaftoside (LOT: 000046201) were obtained from Biosynth (Bratislava, Slovakia). All the standards used were of analytical or HPLC grade. Milli-Q purified water (Merck Millipore, Bedford, MA, USA) was used to prepare all aqueous solutions.

### 2.2. Germination Method

Before germination, seeds were prepared by removing foreign materials and damaged kernels. Seeds were soaked in water at 20 °C (seed-to-water ratio of 1:5 (*w*/*v*)). The soaking water was replaced every hour during the air-rest period to reduce the risk of microbial contamination. After soaking, seeds were laid in a thin layer on perforated metal trays. The trays were placed in a growth chamber with a humidifier to ensure high air humidity (relative humidity, >95%) and germinated at 20 °C. The sprouts were harvested at 72 h after the start of soaking. Before the incorporation of the seeds into bread dough, to prevent undesirable effects, such as overactivity of enzymes, and to ensure microbial safety, seeds were packed in 500 g batches and heat-treated for 40 min at 80 °C.

### 2.3. Preparation of Bread Samples

The control bread formulation was prepared using a straight dough method under controlled laboratory conditions in accordance with national legislation and good manufacturing practices. The detailed formulations of all the experimental bread samples are presented in Table 1. The breads were coded according to the type and addition level of germinated grains: control, S30 and S60 (30% and 60% germinated spelt), O30 and O60 (30% and 60% germinated oat), B30 and B60 (30% and 60% germinated buckwheat), and MG30 and MG60 (30% and 60% multigrain formulations). The ingredients were mixed in a spiral mixer (Diosna SP12, Diosna Dierks & Söhne GmbH, Osnabrück, Germany) for 3 min at low speed and 12 min at high speed. After mixing, the dough rested in the mixer bowl for 30 min. The dough was then divided into 320 g pieces, placed into moulds, and transferred to a fermentation chamber (Gostol-Gopan FK, Nova Gorica, Slovenia) for 45 min at 28 °C and 76% relative humidity. Baking was carried out in a deck oven (Miwe Aero CS, Miwe Michael Wenz GmbH, Arnstein, Germany) for 5 min at 230 °C with steam injection, followed by 20 min at 190 °C and 4 min at 200 °C.

### 2.4. Dietary Fibre Content

The dietary fibre content was determined using the enzymatic–gravimetric method described in AOAC 991.43. The method used three enzymes—heat-stable α-amylase, a protease, and an amyloglucosidase (all enzymes, Cat. No. 112979; Merck KGaA, Darmstadt, Germany)—to hydrolyse the samples under different conditions. The dietary fibre fractions were obtained as indigestible residues after the enzymatic digestion of the non-dietary fibre component. The insoluble dietary fibre was recovered by filtration, and the soluble dietary fibre was precipitated from the filtrate using 96% ethanol. The residual ash and protein contents were determined in the fibre residues to correct the data. The total dietary fibre is defined as the sum of the insoluble and soluble dietary fibre content.

### 2.5. Extraction of Free Phenolic Compounds

Samples of dough and bread were frozen in liquid nitrogen and milled in a laboratory mill (A11 Basic; IKA-Werke, Staufen, Germany). Free phenolics were extracted with 70% (*v*/*v*) aqueous methanol. Briefly, 1 g of milled sample was combined with 3 mL of extraction solvent and the mixture was shaken in the dark at room temperature for 40 min at 200 rpm (EV-403; Tehtnica Železniki, Slovenia). After centrifugation at 8709× *g* for 8 min at 10 °C (Avanti JXN-26; Beckman Coulter, Krefeld, Germany), the supernatant was collected. The extraction procedure was repeated twice, and the combined extracts were adjusted to a final volume of 10 mL with extraction solvent. After filtration through 0.45 μm syringe filters (Chromafil A-45/25; cellulose acetate, hydrophilic membrane; Macherey-Nagel, Düren, Germany), extracts were kept at 2 °C and analyzed for total phenolic content (TPC) and antioxidant activity (AA) within 24 h.

### 2.6. Extraction of Bound Phenolic Compounds

The residues remaining after methanolic extraction were hydrolyzed with sodium hydroxide, as described previously [3]. The solid material was treated with 20 mL of 2 M NaOH and shaken for 4 h at room temperature at 200 rpm (Tehtnica Železniki EV-403, Slovenia). After hydrolysis, the mixture was acidified to pH 3.2–3.4 using concentrated formic acid and centrifuged at 8709× *g* for 8 min at 10 °C (Avanti JXN-26; Beckman Coulter, Krefeld, Germany). The supernatant was filtered through 0.45 µm syringe filters (Chromafil A-45/25; cellulose acetate, hydrophilic membrane; Macherey-Nagel, Düren, Germany) and kept at 2 °C until the analysis of TPC and AA within 24 h.

### 2.7. Total Phenolic Content (TPC)

Total phenolics were determined using the Folin–Ciocalteu method as previously described with minor adjustments [3]. An aliquot of extract (100 µL) was mixed with 1.3 mL of Milli-Q water and 0.3 mL of diluted Folin–Ciocalteu reagent (1:2, *v*/*v*). After 5 min, 0.3 mL of 20% (*w*/*v*) Na_2_CO_3_ was added. The reaction mixture was incubated for 60 min at room temperature, and the absorbance was measured at 765 nm (UV-Vis spectrophotometer; Model 8453; Agilent Technologies, Santa Clara, CA, USA). The results were calculated from a Trolox calibration curve and are expressed as mg Trolox equivalents per g dry weight (mg TE/g DW).

### 2.8. DPPH Radical-Scavenging Activity

The radical-scavenging capacity was evaluated using the DPPH assay according to Živković et al. [3], with slight modifications. Extract (50 µL) was mixed with 250 µL of acetate buffer and methanol to a final volume of 1 mL. Finally, 1 mL of 0.2 mM DPPH in methanol was added. After incubation for 1 h in the dark, the absorbance was recorded at 517 nm (UV-Vis spectrophotometer; Model 8453; Agilent Technologies, CA, USA). Antioxidant activity was quantified using a Trolox standard curve and is expressed as mg TE/g DW.

### 2.9. Purification of Extracts

Prior to LC–MS/MS analysis, extracts were purified using 100 mg Strata-X RP cartridges (Phenomenex, Torrance, CA, USA). Cartridges were conditioned with methanol followed by water. Diluted free extracts (extract:water 1:9, 30 mL) or 3 mL volumes of hydrolyzed extracts were loaded onto the cartridges. After washing with water and vacuum drying, the retained compounds were eluted with 2 mL of 70% aqueous methanol. The eluates were filtered through 0.20 μm syringe filters and stored at −80 °C until analysis.

### 2.10. Liquid Chromatography–Mass Spectrometry Analysis

To determine the concentrations of individual compounds, reversed-phase LC-MS/MS analysis was used for separation and quantification. The LC system used was an ACQUITY™ UPLC™ H-Class PLUS System (Waters, Milford, MA, USA) coupled with a Xevo TQ-S micro triple quadrupole mass spectrometer (Waters, Milford, MA, USA). Chromatographic separation was carried out using a C18 column (2.7 μm, 150 mm × 2.1 mm; Ascentis Express) with a C18 guard column (2.7 μm, 5 mm × 2.1 mm; Ascentis Express; Supelco, Bellefonte, PA, USA). The conditions used were as follows: column temperature, 35 °C; injection volume, 2 µL; and mobile phase flow rate, 320 µL/min. The components of the mobile phase were 0.1% aqueous formic acid (solution A) and acetonitrile (solution B). The mobile phase gradient was programmed as follows (%B): 0–4 min, 10%; 4–18 min, 10–60%; 18–18.2 min, 60–80%; 18.2–20 min, 80%; 20–20.2 min, 80–10%; 20.2–26 min, 10%. Detection was performed with scanning diode array spectra from 240 nm to 650 nm.

The mass spectrometer was operated in negative ionization mode, and the operating conditions were as follows: electrospray capillary voltage, 3.5 kV; cone voltage, 20 V; extractor voltage, 2 V; source block temperature, 100 °C; desolvation temperature, 350 °C; cone gas flow rate, 30 L/h; and desolvation gas flow rate, 350 L/h. The data signals were acquired and processed on a PC using MassLynx software (V4.2 2019; Waters Corporation). With comparison against previously determined calibration curves, the individual compounds were identified by comparing their retention times and both the spectroscopic and mass spectrometric data, with quantification according to peak areas.

### 2.11. Bread Properties

#### 2.11.1. Specific Volume

The specific volume of prepared bread was evaluated by the rapeseed displacement method (AACC method 10-05.01). The results are expressed as the ratio between bread volume and weight (mL/g).

#### 2.11.2. Colour Measurement of Crust

The colour of bread crust was determined by measuring three colour properties (L∗, a∗, and b∗) using a Minolta CR-400 device (Konica Minolta, Kyoto, Japan). L∗ represents luminosity (black at 0 and white at 100); a∗ indicates the green–red spectrum, with negative values indicating green and positive values indicating red; and b∗ represents the blue–yellow spectrum, with negative values indicating blue and positive values indicating yellow. The colour was determined by taking five measurements across the crust surface and averaging the values.

#### 2.11.3. Texture Analysis

The texture of bread samples was evaluated according to AACC Method 74-09. Measurements were carried out using a TA-XT Plus Texture Analyser (Stable Micro Systems Ltd., Godalming, UK) equipped with a 36 mm cylindrical probe. Bread samples were sliced into 25 mm thick slices, and the crumb texture was analyzed using a compression test at 40% deformation. Texture measurements were carried out 3 h after baking (day 0), as well as after 24 h and 48 h of storage, in order to monitor changes in textural properties during storage. The analysis was performed under controlled conditions using a compression test, and the resulting force–time curves were used to evaluate textural parameters. All measurements were performed in triplicate.

#### 2.11.4. Sensory Evaluation

Sensory evaluation of the bread samples was carried out 3 h after baking, by a trained expert panel consisting of five assessors with prior experience in bakery product evaluation and descriptive sensory analysis. The evaluation was conducted as an analytical quality assessment rather than a consumer preference study. Each participant received representative slices approximately 2 cm thick, which were coded to ensure unbiased assessment. The aim of the evaluation was to determine the overall acceptability of breads enriched with germinated seeds. The assessment was based on a 20-point scale, with different attributes contributing proportionally to the total score. The evaluated parameters included shape and general appearance (1 point), crust properties and appearance (2 points), crumb appearance (4 points), structure and elasticity (4 points), and aroma and taste (9 points). Panellists examined the external characteristics of the bread, focusing on its shape and the appearance and colour of the crust. The bread was then sliced to allow evaluation of the crumb, where the colour, porosity, and uniformity of the porosity were observed. In addition, the texture, elasticity of the crumb, and aroma and taste of each sample were evaluated. Each attribute was rated according to its intensity and quality compared to the control bread.

### 2.12. Statistical Analysis

All experiments were carried out three times using a complete randomization method. All spelt extracts were prepared in duplicate. Data are reported as the mean +/− standard deviation (SD) for three analyses for each extract. The results were subjected to two-way ANOVA, and the significances of the differences between the mean values were determined using Tukey’s multiple comparison tests. All of the tests were performed using SPSS Statistics software (version 24; IBM, New York, NY, USA). Statistical significance was defined at the level of *p* < 0.05.

## 3. Results and Discussion

### 3.1. Effect of Germination on Total Phenolic Content and Antioxidant Activity

**Figure 1:**
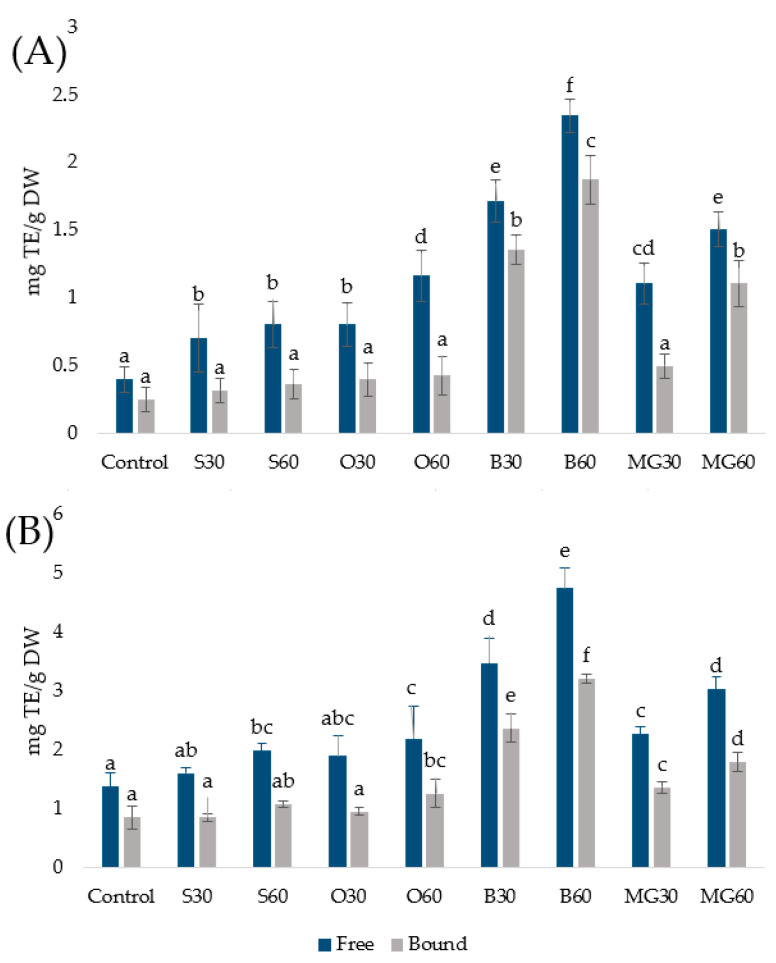
Total phenolic content (**A**) and antioxidant activity (**B**) in free and bound fractions of breads enriched with germinated grains. Data are means ± SDs from three independent replicates. Means with different letters indicate statistically significant differences between the bread samples (*p* < 0.05).

The total phenolic content (TPC) and antioxidant activity (AA) of methanolic extracts of all the bread samples were determined using the Folin–Ciocalteu (FC) and DPPH assays, respectively. The results are expressed as Trolox equivalents (mg TE g^−1^ dry matter) and presented in Figure 1.

Both assays revealed that breads enriched with germinated grains exhibited significantly higher phenolic content and antioxidant activity compared to the control, confirming that grain germination enhances the accumulation of bioactive metabolites such as phenolic compounds [14]. The increase in phenolic compounds and AA was generally proportional to the level of germinated grains added to the dough, further emphasizing their role as a natural source of antioxidants.

Among the studied grains, germinated buckwheat resulted in the most pronounced increase in both TPC and AA. This is consistent with previous studies reporting that buckwheat possesses one of the highest intrinsic phenolic potentials among pseudocereals and that germination enhances the accumulation of rutin, quercetin derivatives, and phenolic acids [3,15]. In the present study, the increase is evident not only in the free fraction but also in bound phenolics, suggesting that germination and subsequent baking conditions facilitate the partial release of phenolics associated with cell wall components. The total TPC of B60 reached nearly 8 mg TE g^−1^, representing more than a threefold increase compared to the control. It should be noted that, in our previous studies on individual germinated grains [2,3,4], germinated buckwheat consistently exhibited substantially higher TPC and AA compared to germinated spelt and oat. The pronounced effect observed in the B30/B60 breads confirms the effective transfer of these compounds into the baked product. The rise in bound phenolics is of particular nutritional importance because these compounds are known to persist through digestion and exert antioxidant effects in the colon, where they may contribute to gut health-related benefits [16].

Germinated oats also contributed substantially to the enhancement of TPC and AA. Compared with other cereals, oats are rich source of avenanthramides—oat-specific phenolic alkaloids with strong radical-scavenging capacity. The O60 sample displayed approximately 55% higher total TPC than the control, indicating that oat germination effectively boosts the antioxidant potential of the final baked product.

Germinated spelt produced smaller but consistent improvements, likely due to its compositional similarity to wheat [17], where the phenolic profile is dominated by ferulic acid and *p*-coumaric acids mostly in bound form. The increase in free phenolics was modest, while the rise in bound phenolics indicates enhanced accumulation of phenolic compounds in newly formed tissues during germination. These findings are consistent with our earlier report showing moderate but nutritionally relevant increases in spelt phenolics after germination [2].

Multigrain breads also exhibited significantly elevated TPC and AA compared to the control. The MG60 sample showed more than a twofold increase in TPC and approximately a fourfold increase in AA compared to the control. Although the effect was less pronounced than in buckwheat-enriched breads, the combined use of different germinated grains likely contributed to a broader spectrum of secondary metabolites.

Pearson correlation analysis demonstrated a very strong positive relationship between total phenolic content (FC assay) and antioxidant activity (DPPH assay) across all bread samples (r = 0.92, *p* < 0.001, *n* = 72). This result confirms that germination-induced increases in phenolic compounds were the primary factor responsible for the enhanced antioxidant activity, although compound-specific reactivity differences may partly explain variations among individual grain types. Although the overall trends obtained with FC and DPPH methods were similar, absolute DPPH values were lower, probably reflecting differences in the selectivity and reactivity of individual antioxidants. It is important to recognize the limitations of in vitro antioxidant assays because different bioactive compounds may have distinct reactivities toward the FC and DPPH reagents (Table S1); therefore, variations between the signals obtained by these two assays can be explained by differences in the chemical nature of the compounds present in the extracts. Nevertheless, the strong correlation between TPC and AA observed in this study is consistent with our previous studies [2,3,4] and clearly indicates that the incorporation of germinated grains enhances the functional and nutritional value of bread.

### 3.2. Characterization of Secondary Metabolites by Liquid Chromatography–Mass Spectrometry

**Figure 2:**
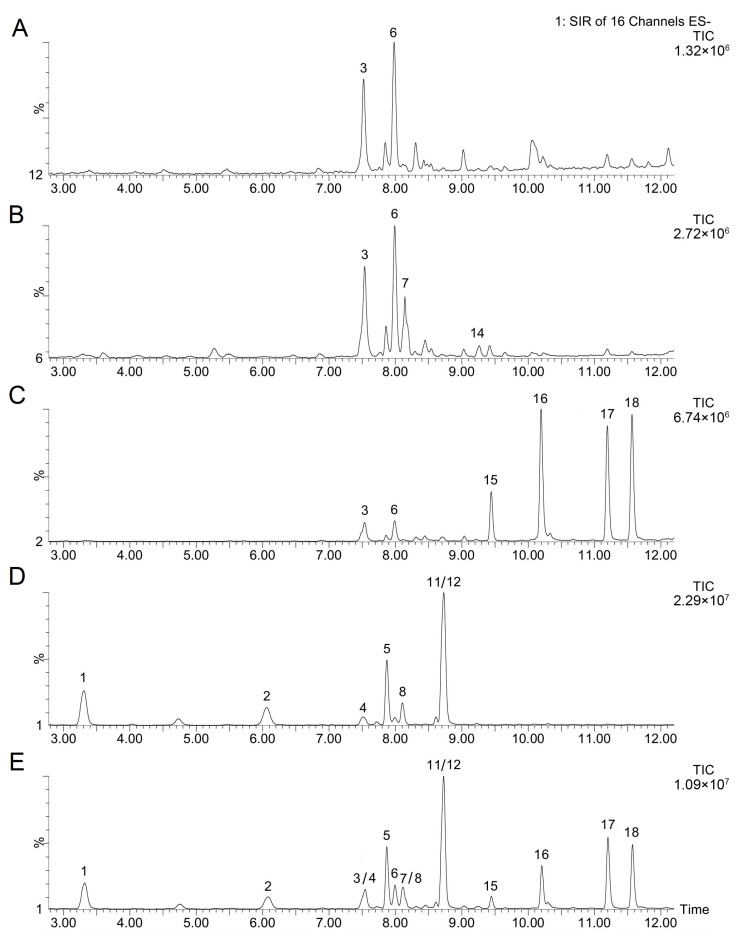
Chromatograms obtained through LC–MS/MS analysis of the free fraction of control bread (**A**), S60 (**B**), O60 (**C**), B60 (**D**) and MG60 (**E**). Peaks: 1, catechin; 2, epicatechin; 3, trans-*p*-coumaric acid; 4, schaftoside isomer; 5, isoorientin; 6, schaftoside; 7, orientin; 8, BOA; 11, vitexin; 12, rutin; 14, MBOA; 15, unidentified; 16, AVN C; 17, AVN A; 18, AVN B (peaks: 9, cis-*p*-coumaric acid; 10, trans-ferulic acid; 13, cis-ferulic acid; were present only in bound fraction).

The LC-MS/MS analysis provided a detailed quantification of individual phenolic compounds and benzoxazinoids in both free and bound fractions of the bread samples (Table 2, Table 3 and Figure 2). The results demonstrate that the incorporation of germinated grains diversified and intensified the phytochemical profile of the bread. The type and concentration of bioactive compounds directly corresponded to the specific grain used and increased proportionally with the level of addition.

In breads enriched with germinated spelt, benzoxazinoids were the predominant compounds specific to this grain. 2-Benzoxazolinone (BOA) and 6-methoxy-2-benzoxazolinone (MBOA) were detected only in spelt-containing samples, with their concentrations increasing in a dose-dependent manner. The free fraction of the S60 bread contained 13.52 ± 0.90 µg/g DW of BOA and 4.02 ± 0.53 µg/g DW of MBOA. Benzoxazinoids are secondary metabolites found in some cereals, including spelt and wheat; in nongerminated grains, they are present at very low or undetectable levels; therefore, they were not detected in the control bread, which is consistent with our previous findings [2]. The content of schaftoside and its isomer was also elevated in comparison with the control. These compounds were also transferred to the multigrain breads, confirming the contribution of spelt to the overall phytochemical pool. The presence of these compounds aligns with our previous findings on germinated spelt [2]. In addition to the free fraction of secondary metabolites, the bound phenolic content, mainly ferulic acid (both cis- and trans-isomers) and trans-*p*-coumaric acids, was substantially higher in the spelt-enriched breads compared to the control, with the S60 sample showing the highest values (190.71 ± 24.03 µg/g DW; 38.39 ± 2.64 µg/g DW; and 5.70 ± 0.73 µg/g DW, respectively).

The breads containing germinated naked oat were characterized by the presence of avenanthramides A, B, and C. These compounds, unique to oats, were found in high amounts in the free fraction, increasing proportionally with the level of oat addition. The O60 bread contained 12.38 ± 0.59 µg/g DW of AVN A; 20.54 ± 1.21 µg/g DW of AVN B; and 18.55 ± 1.91 µg/g DW of AVN C. The successful integration of these compounds into the bread matrix highlights the potential of using germinated naked oats to enhance the functional value of bakery products. The multigrain breads also contained measurable amounts of all three avenanthramides, with MG60 containing 5.14 ± 0.27 (AVN A); 7.54 ± 0.46 (AVN B); and 6.50 ± 0.44 (AVN C) µg/g DW, demonstrating the effective contribution of oats even in a blended formulation.

The most diverse and abundant profile of secondary metabolites was found in breads with germinated buckwheat. The free fraction of the B60 bread was rich in rutin (35.11 ± 3.10 µg/g DW), vitexin (41.99 ± 1.88 µg/g DW), orientin (20.82 ± 1.12 µg/g DW), and isoorientin (23.74 ± 1.21 µg/g DW). Epicatechin, which is significantly elevated during buckwheat germination [3], was also detected at 18.24 ± 1.88 µg/g DW in the B60 sample. The high concentration of these compounds confirms that the germination process effectively enhances the flavonoid content, and that a substantial portion survives the thermal processing during baking. The bound fraction of buckwheat breads contained smaller but detectable amounts of orientin, isoorientin, and vitexin.

The multigrain breads exhibited a composite phytochemical profile, containing benzoxazinoids from spelt, avenanthramides from oats, and flavonoids from buckwheat. This demonstrates the complementary effect of using a grain mixture, resulting in a bread with a wider profile of secondary metabolites. The MG60 bread contained BOA (5.42 µg/g DW), AVN B (7.54 µg/g DW), and rutin (11.81 µg/g DW) simultaneously. Such diversity likely broadens the spectrum of antioxidant mechanisms, as different phenolic classes contribute distinctly to radical scavenging, reducing capacity, and potential synergistic interactions. The control bread showed a much simpler profile, with low background levels of compounds like schaftoside and its isomer, and ferulic and *p*-coumaric acids.

Comparison of the LC–MS/MS profiles of dough samples prior to baking (Supplementary Table S2) and the corresponding bread samples showed comparable secondary metabolite compositions, indicating that the applied conditions did not substantially affect the presence of the identified compounds. The majority of bioactive compounds remained detectable at comparable concentrations after baking. For example, rutin, vitexin, orientin, and isoorientin in buckwheat-enriched samples showed only moderate decreases, suggesting considerable resistance to thermal degradation. Similarly, avenanthramides A, B, and C in oat-enriched breads were largely preserved. The minor changes observed for phenolic acids may reflect both partial degradation and simultaneous release from bound forms during thermal processing. These results indicate that the bread-making process does not substantially compromise the stability of secondary metabolites, supporting the feasibility of using germinated grains in functional bakery products.

In summary, the LC-MS/MS analysis confirmed that grain-specific bioactive compounds are successfully incorporated into bread upon enrichment with germinated seeds and that the concentration of most compounds is dose-dependent. The stability of these compounds during bread-making is crucial for developing functional foods with enhanced nutritional value.

### 3.3. Dietary Fibre Content

The results for the dietary fibre content of the bread samples are presented in Table 4. The incorporation of germinated grains significantly increased the total dietary fibre (TDF) content compared to the control bread. The control bread, formulated with white wheat flour (T500), had a TDF content of 2.78 g/100 g, which is typical for conventional white bread [18].

The addition of 30% germinated grains increased the TDF to a range of 4.05–4.32 g/100 g, representing an increase of approximately 53–55% over the control. The 60% enrichment level resulted in a further significant rise in TDF, with values ranging from 4.96 to 5.16 g/100 g. This corresponds to an increase of 78–86% compared to the control, bringing the fibre content to a level comparable to or exceeding that of many wholemeal breads.

This enhancement was driven by increases in both the insoluble (IDF) and soluble (SDF) dietary fibre fractions. The IDF content rose from 2.16 g/100 g in the control to a maximum of 3.59 g/100 g in the Spelt 60 sample. This is consistent with the high bran content of the whole, germinated seeds, which are a rich source of insoluble fibres such as cellulose and lignin. The SDF fraction also increased substantially, from 0.62 g/100 g in the control to over 1.80 g/100 g in the Oat 60 sample—a nearly three-fold increase. The rise in SDF can be attributed to components like beta-glucans from oats and spelt, as well as arabinoxylans and other soluble polysaccharides released or synthesized during the germination process.

The breads enriched with germinated spelt showed the highest levels of IDF, while those with germinated oats and buckwheat showed the highest SDF content. The multigrain breads exhibited a balanced fibre profile, effectively combining the contributions of all three grain types. The SDF/TDF ratio also increased from 22% in the control to approximately 30–36% in most enriched samples, indicating a relative enrichment of soluble fibre, which is particularly beneficial for its prebiotic and blood glucose-regulating properties [19].

### 3.4. Bread Properties

In Table 5 are summarized all the analyzed bread properties, including specific loaf volume, water content, crust colour parameters, texture characteristics, and sensory evaluation scores. These results enable a direct comparison between the control sample and breads enriched with spelt, oat, buckwheat, and multigrain flours at two substitution levels (30% and 60%).

#### 3.4.1. Specific Volume

**Figure 3:**
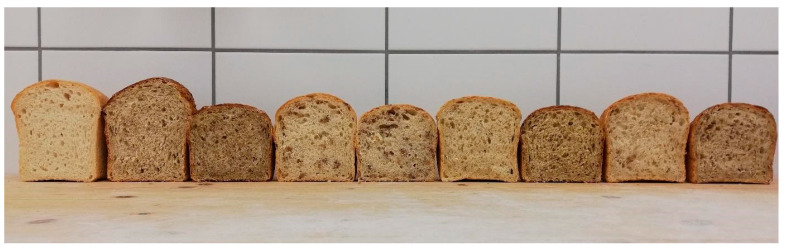
Cross-sections of prepared breads. From left to right: Control, B30, B60, S30, S60, O30, O60, MG30, MG60.

The specific volume of bread is an important indicator of product quality that reflects crumb porosity and the degree of dough expansion during baking (Figure 3). The control bread exhibited the highest specific volume among all samples, averaging 3.42 mL/g. Among the breads enriched with 30% germinated grains, most samples showed only a moderate reduction in specific volume, and a statistically significant decrease was confirmed only for the bread containing germinated spelt. Breads with 60% germinated grain addition displayed significantly lower specific volumes than the control. The most pronounced decrease was observed in the bread enriched with 60% germinated oats (2.63 mL/g).

The results indicate that the incorporation of germinated grains negatively affects the specific volume of bread, and that this effect increases with higher substitution levels. Several factors likely contribute to these differences, including the physicochemical characteristics of the germinated grains, the content of secondary metabolites, and potential enzymatic activity. Phenolic compounds can interact with thiol groups in gluten proteins, disrupting the development of the protein network and consequently reducing the specific volume [20]. Dietary fibre, abundant in germinated grains, can influence the specific volume, leading to an underdeveloped gluten network or a dilution effect that weakens gas retention during proofing [21]. Increased enzymatic activity in germinated grains may also contribute to the reduction in specific volume. During germination, hydrolytic enzymes such as amylases and proteases accumulate [22], promoting starch and protein degradation. As a result, doughs containing germinated grains are often weaker and stickier, making them difficult to handle and leading to reduced loaf volume. According to Marti et al. [22], controlled germination and limited enzyme activity can positively affect bread fermentation; however, in the present study, the germinated grains were thermally treated prior to use to ensure the thermal inactivation of hydrolytic enzymes. Although insufficient heat treatment might partially preserve enzyme activity, pasteurization at 80 °C for 40 min was considered sufficient for complete inactivation. Therefore, the most plausible explanation for the reduced specific volume of breads containing germinated grains is the altered composition of the raw material and the partial replacement of wheat flour by whole grains. Because whole grains do not participate in dough expansion to the same extent as flour, the effective proportion of gluten-forming material was reduced.

#### 3.4.2. Colour of Bread Crust

The colour parameters (L*, a*, and b*) of the bread crusts are presented in Table 5. In general, all breads containing germinated grains—except for the S30 sample—exhibited significantly darker crusts than the control, as indicated by their noticeably lower L* values compared to the control average (L* = 70.17). Samples with a lower proportion of germinated grains displayed lighter crust coloration. Among all the samples, the B60 bread showed the darkest crust (L* = 48.03), being significantly darker than most other samples.

The red–green component (a*) also differed considerably between the samples. The control bread displayed the lowest a* value (6.22), indicating the least reddish hue. All enriched samples exhibited higher a* values, demonstrating intensified red coloration. This effect was especially prominent in the B60 sample (a* = 12.41), followed closely by O60 and S60. The consistent rise in a* with increasing germinated grain content suggests that either intensified browning reactions or inherent plant pigments contributed to the enhanced reddish tone. The b* parameter, representing the yellow–blue axis, was positive for all crusts, indicating yellowish coloration. Samples enriched with germinated grains generally showed slightly lower b* values than the control (32.38), meaning a weaker yellow component. This was most evident in breads containing germinated buckwheat, where b* values decreased to 27.56 (B60).

Crust colour is influenced by multiple factors, including baking conditions, dough composition, and the concentration of precursors participating in colour-forming reactions [23]. The darkening of the crust is primarily caused by polymerization reactions producing brown pigments through Maillard and caramelization reactions. Enhanced hydrolytic enzyme activity during germination leads to the breakdown of starch and proteins into soluble forms [22,24], thereby providing a greater pool of precursors for browning reactions. Marti et al. [22] also reported that the addition of germinated wheat flour contributed to darker crust coloration, with increases observed in all three colour parameters. In our study, although a similar pattern of crust darkening was observed, an increase in the b* parameter was not confirmed.

In addition to Maillard and caramelization reactions, natural plant pigments may also contribute to the darker crust colour observed in certain samples. This effect was particularly pronounced in breads containing germinated buckwheat, which is known to accumulate large amounts of flavonoids during germination [3], giving the crust a reddish-brown appearance.

#### 3.4.3. Texture Analysis

The textural quality of bread is one of the most important attributes determining consumer acceptance, and crumb firmness is widely recognized as the key indicator of freshness and overall bread quality. The results of texture analysis are summarized in Table 5.

On the day of baking, crumb hardness differed distinctly among formulations. The control bread was the softest (4.55 N), whereas the addition of germinated grains generally produced firmer crumbs. The highest hardness at 3 h after baking was observed in the bread containing 60% germinated spelt, which also had one of the lowest specific volumes. This relationship between specific volume and hardness was evident across several samples: denser loaves such as O60 and P60 exhibited higher firmness, whereas breads with larger loaf volumes—particularly the control and B30—were softer. This supports the established principle that specific volume is closely linked to textural softness, as less-expanded crumb structures resist compression more strongly [25].

After 24 h of storage, hardness increased in all samples, but the relative differences among formulations changed (Figure S1). The control bread, which initially had the lowest firmness, became harder than breads enriched with 30% germinated buckwheat and the multigrain formulations. This indicates that, although germinated grains may initially produce a firmer crumb due to the physical presence of seed particles and altered dough rheology, they may also slow firming during storage. After 48 h, the MG60 bread was the softest of all samples, despite its lower specific volume and higher initial hardness.

The staling trend can be explained by various mechanisms: starch retrogradation, water migration from gluten to starch, and the formation of a progressively more rigid gluten–starch network [26,27]. As the germinated grains used in this study were pasteurized, differences in firming rates cannot be attributed to enzymatic activity such as amylase-mediated starch hydrolysis, which is sometimes responsible for reduced staling in non-pasteurized germinated grains [28]. A more plausible mechanism is their influence on water distribution: germinated grains typically contain structural elements capable of binding and retaining water, thereby modifying moisture migration during storage and limiting crumb firming. This is supported by the generally higher water contents of enriched bread compared to the control.

#### 3.4.4. Sensory Evaluation

Sensory analysis was performed by a trained panel of five assessors on bread samples cooled to room temperature. The results of the sensory evaluation are presented in Table 5. The evaluation followed the system of the Bakery Association of the Chamber of Commerce and Industry of Slovenia and included the assessment of shape and appearance, crust properties, crumb appearance, crumb structure and elasticity, and aroma and taste, supported by descriptive comments.

For the shape and appearance, most samples—including all breads with 30% germinated grain addition—received the maximum score. Only S60 scored slightly lower, reflecting its smaller loaf volume and reduced proofing, consistent with specific volume measurements. Nevertheless, no structural defects such as collapse or excessive cracking were observed.

Crust appearance and properties varied moderately among samples. The control bread received the highest rating due to its smooth, glossy crust, whereas B30, B60, S60, and O60 scored slightly lower. Darker coloration and, in some cases, visible germinated seeds were typical for breads with germinated grains, particularly those with buckwheat and oats. Wrinkling (S60, O60) and dull or cracked surfaces (B60, B30) were occasionally noted. These changes corresponded well with instrumental colour data showing darker and less yellow crusts in breads with higher germinated grain content. The darker tones were expected, as germination increases free sugars and enhances non-enzymatic browning.

Crumb appearance showed more pronounced differences. Samples B30, O30, and MG30 achieved the maximum score, while S60 and O60 were significantly lower. The most common defect was localized pore compaction near loaf edges, likely due to uneven heat transfer during baking. Lower substitution levels generally produced lighter, more open crumbs, whereas 60% additions—particularly buckwheat and oat—resulted in denser structures. Despite these differences, all enriched breads displayed acceptable overall crumb morphology with no evidence of structural collapse or water rings.

Crumb structure and elasticity produced the largest spread of scores. Control, S30, B30, and O30 achieved the maximum, whereas all 60% samples (except S60) and both MG samples scored significantly lower. Stickiness was the main negative attribute, especially in B60, O60, and MG60, but also present to a milder degree in B30 and O30. This was likely linked to the high water-binding capacity of dietary fibre and residual enzymatic activity in germinated grains, conditions known to cause moist, sticky crumbs in whole-grain breads [29,30]. In germinated spelt breads, some panellists detected hard dried grain particles, which became more apparent with higher additions. Despite these attributes, elasticity was generally good, and crumbs typically recovered their shape after compression.

Aroma and taste were positively affected by germinated grain addition. With the exception of P60, all enriched samples received equal or higher scores than the control. Characteristic aromas were noted across samples: sprout-like “green” notes in buckwheat breads, slight sweetness in O60 and A60, and malty–nutty tones in spelt and oat breads. MG60 exhibited a strong buckwheat-dominant aroma, while P30 and O30 were described as mild. No foreign or off-odours were detected, and all samples displayed clean bread-like flavour profiles.

The total sensory scores confirmed high overall acceptability across all breads. The highest scores (19.5 points) were recorded for A30 and A30/B30, followed by MG30, P30, and the control. Among the 60% formulations, MG60 performed best (19.0 points), while P60 scored lowest (18.2 points), largely due to its darker crust colour, denser crumb, and more intense sprout-like aroma. Despite these differences, all samples scored above 18 points, classifying them as products of very good sensory quality.

## 4. Conclusions

Based on our study of various bread samples enriched with 30% or 60% of germinated spelt, buckwheat, naked oat, or their mixture, bread fortification is highly feasible. The incorporation of germinated seeds significantly influenced the chemical composition and quality characteristics of white wheat bread. The results enabled several conclusions regarding nutritional, technological, and sensory properties. The nutritional upgrade of wheat control bread included various species-specific secondary metabolites, such as benzoxazinoids (BOA, MBOA), avenanthramides (AVN A, B, C), flavonoids (e.g., rutin, vitexin, orientin), and others present in the samples, with multigrain bread containing the full set of metabolites in significant quantities. In the bound fraction, ferulic and *p*-coumaric acids were dominant compounds across samples. The total phenolic content (TPC) and antioxidant activity (AA) were markedly increased in the enriched breads, with germinated buckwheat providing the most pronounced increase. Increasing the incorporation level from 30% to 60% significantly influenced the concentration of secondary metabolites and dietary fibre. However, higher substitution levels also affected certain technological parameters, including reduced specific loaf volume and more pronounced crust coloration. Despite minor technological deviations, sensory evaluation confirmed good overall acceptance of all enriched bread variants. Textural analyses also showed the significant impact of adding germinated seeds on slowing the bread staling rate during storage. The results demonstrate that germinated grain incorporation represents a promising strategy for improving the functional value of bakery products while maintaining acceptable technological and sensory quality. Considering both nutritional and technological aspects, a 30% incorporation level appears to represent the optimal compromise for practical application, ensuring substantial enhancement of secondary metabolite and dietary fibre content while maintaining high overall bread quality. Higher substitution levels (60%) may be suitable for specialized functional bakery products in which nutritional maximization is prioritized. From a commercial perspective, germination introduces an additional processing step that may increase production time and operational complexity. Nevertheless, the enhanced phytochemical profile and added functional value may justify these costs, particularly within the growing market for functional and premium baked products. Future research should further investigate the optimization of substitution levels, long-term storage stability, and process standardization to facilitate industrial-scale application.

## Figures and Tables

**Table 1:** Formulation of bread samples enriched with germinated grains. Enrichment levels (30% and 60%) represent germinated seed addition percentages on a flour weight basis.

Sample Code	Wheat Flour (g)	Water (mL)	Germinated Seeds (g)	Salt (g)	Yeast (g)
Control	1000	675	0	19	7
S30	1000	675	300	19	7
S60	1000	675	600	19	7
O30	1000	675	300	19	7
O60	1000	675	600	19	7
B30	1000	675	300	19	7
B60	1000	675	600	19	7
MG30	1000	675	300	19	7
MG60	1000	675	600	19	7

**Table 2:** Concentrations of free phenolic compounds and benzoxazinoids (µg/g DW) in control bread and breads enriched with 30% or 60% germinated spelt, oat, and buckwheat. Data are means ± SDs from three independent replicates. Means with different letters in rows indicate statistically significant differences between the bread samples (*p* < 0.05). ND—compound not detected.

Phenolic Compound	Content of Benzoxazinoids and Phenolics (µg/g DW) in Samples of Bread								
	Control	S 30	S 60	O 30	O 60	B 30	B 60	MG 30	MG 60
Free Fraction									
AVN A	ND	ND	ND	7.73 ± 0.90 ^b^	12.38 ± 0.52 ^c^	ND	ND	2.92 ± 0.30 ^a^	5.14 ± 0.27 ^b^
AVN B	ND	ND	ND	11.62 ± 1.08 ^c^	18.75 ± 1.21 ^d^	ND	ND	3.67 ± 0.35 ^a^	7.54 ± 0.46 ^b^
AVN C	ND	ND	ND	10.73 ± 0.76 ^c^	18.55 ± 1.91 ^d^	ND	ND	3.73 ± 0.38 ^a^	6.5 ± 0.44 ^b^
BOA	ND	7.86 ± 0.77 ^c^	13.52 ± 0.9 ^d^	ND	ND	ND	ND	3.42 ± 0.27 ^a^	5.42 ± 0.57 ^b^
MBOA	ND	2.05 ± 0.26 ^c^	4.02 ± 0.53 ^d^	ND	ND	ND	ND	0.78 ± 0.15 ^a^	1.49 ± 0.14 ^b^
Orientin	ND	ND	ND	ND	ND	12.32 ± 1.68 ^c^	20.82 ± 1.12 ^d^	4.22 ± 0.79 ^a^	7.05 ± 0.57 ^b^
Isoorientin	ND	ND	ND	ND	ND	13.39 ± 1.46 ^c^	23.74 ± 1.21 ^d^	5.08 ± 0.65 ^a^	8.62 ± 0.59 ^b^
Rutin	ND	ND	ND	ND	ND	19.81 ± 2.02 ^c^	35.11 ± 3.10 ^d^	7.60 ± 0.86 ^a^	11.81 ± 0.54 ^b^
Vitexin	ND	ND	ND	0.27 ± 0.04 ^a^	0.52 ± 0.11 ^b^	27.71 ± 2.26 ^e^	41.99 ± 1.88 ^f^	9.50 ± 0.48 ^c^	17.69 ± 0.56 ^d^
Catechin	ND	ND	ND	ND	ND	17.59 ± 1.86 ^c^	32.07 ± 3.02 ^d^	5.61 ± 0.72 ^a^	9.26 ± 0.53 ^b^
Epicatechin	ND	ND	ND	ND	ND	9.51 ± 1.05 ^c^	18.24 ± 1.88 ^d^	3.11 ± 0.26 ^a^	5.47 ± 0.35 ^b^
Schaftoside	4.00 ± 0.50 ^b^^c^	5.64 ± 0.61 ^e^	7.56 ± 1.19 ^f^	3.63 ± 0.28 ^abc^	3.37 ± 0.23 ^ab^	3.45 ± 0.34 ^ab^	2.99 ± 0.42 ^a^	4.41 ± 0.37 ^cd^	4.78 ± 0.45 ^d^
Schaftoside isomer	3.13 ± 0.11 ^bc^	3.92 ± 0.25 ^d^	5.30 ± 0.91 ^e^	2.78 ± 0.15 ^ab^	2.51 ± 0.17 ^a^	2.82 ± 0.23 ^ab^	2.27 ± 0.17 ^a^	3.50 ± 0.13 ^cd^	3.86 ± 0.22 ^d^
trans-ferulic acid	0.23 ± 0.03 ^a^	0.56 ± 0.09 ^d^	0.73 ± 0.06 ^e^	0.70 ± 0.07 ^e^	1.00 ± 0.13 ^f^	0.30 ± 0.02 ^ab^	0.38 ± 0.05 ^bc^	0.50 ± 0.09 ^cd^	0.61 ± 0.08 ^de^
cis-ferulic acid	0.14 ± 0.04 ^a^	0.21 ± 0.05 ^b^	0.32 ± 0.02 ^c^	0.26 ± 0.02 ^bc^	0.42 ± 0.05 ^d^	0.23 ± 0.04 ^b^	0.28 ± 0.05 ^bc^	0.26 ± 0.06 ^bc^	0.39 ± 0.07 ^d^
trans-*p*-Coumaric acid	0.23 ± 0.03 ^a^	0.57 ± 0.05 ^b^	0.79 ± 0.07 ^cd^	0.69 ± 0.06 ^c^	0.93 ± 0.06 ^e^	1.36 ± 0.12 ^g^	2.15 ± 0.12 ^h^	0.83 ± 0.09 ^de^	1.22 ± 0.07 ^f^

**Table 3:** Concentrations of bound phenolic compounds (µg/g DW) in control bread and breads enriched with 30% or 60% germinated spelt, oat, and buckwheat. Data are means ± SDs from three independent replicates. Means with different letters in rows indicate statistically significant differences between the bread samples (*p* < 0.05). ND—compound not detected.

Phenolic Compound	Content of Benzoxazinoids and Phenolics (µg/G DW) in Samples of Bread								
	Control	S30	S60	O30	O60	B30	B60	MG30	MG60
**Bound Fraction**									
Orientin	ND	ND	ND	ND	ND	0.52 ± 0.07 ^b^	0.96 ± 0.06 ^c^	0.30 ± 0.05 ^a^	0.48 ± 0.04 ^b^
Isoorientin	ND	ND	ND	ND	ND	0.36 ± 0.04 ^b^	0.75 ± 0.09 ^c^	0.20 ± 0.02 ^a^	0.36 ± 0.01 ^b^
Vitexin	ND	ND	ND	ND	ND	0.80 ± 0.09 ^b^	1.56 ± 0.1 ^c^	0.40 ± 0.06 ^a^	0.79 ± 0.09 ^b^
Schaftoside	0.32 ± 0.03 ^b^	0.69 ± 0.05 ^d^	1.06 ± 0.11 ^e^	0.31 ± 0.08 ^ab^	0.23 ± 0.03 ^ab^	0.25 ± 0.05 ^ab^	0.20 ± 0.03 ^a^	0.56 ± 0.08 ^c^	0.66 ± 0.08 ^d^
Schaftoside isomer	0.24 ± 0.05 ^bc^	0.32 ± 0.03 ^d^	0.48 ± 0.06 ^e^	0.18 ± 0.04 ^ab^	0.14 ± 0.03 ^a^	0.13 ± 0.04 ^a^	0.14 ± 0.04 ^a^	0.26 ± 0.05 ^cd^	0.26 ± 0.05 ^cd^
trans-ferulic acid	53.86 ± 4.36 ^a^	125.02 ± 8.65 ^d^	190.71 ± 24.03 ^e^	78.97 ± 6.2 ^b^	108.35 ± 8.18 ^c^	47.46 ± 4.26 ^a^	45.39 ± 3.78 ^a^	86.41 ± 7.51 ^b^	125.59 ± 7.05 ^d^
cis-ferulic acid	16.14 ± 1.88 ^b^	31.05 ± 3.36 ^d^	38.39 ± 2.64 ^e^	22.81 ± 1.68 ^c^	30.68 ± 3.44 ^d^	14.12 ± 1.99 ^ab^	10.98 ± 1.32 ^a^	25.33 ± 1.39 ^c^	29.65 ± 4.35 ^d^
trans-*p*-Coumaric acid	0.91 ± 0.08 ^a^	3.12 ± 0.24 ^b^	5.70 ± 0.73 ^d^	4.35 ± 0.68 ^c^	6.93 ± 0.46 ^e^	1.07 ± 0.11 ^a^	1.47 ± 0.18 ^a^	2.67 ± 0.19 ^b^	4.01 ± 0.3 ^c^
cis-*p*-Coumaric acid	0.19 ± 0.01 ^a^	0.46 ± 0.1 ^b^	0.60 ± 0.15 ^b^	0.87 ± 0.13 ^c^	1.30 ± 0.07 ^d^	0.24 ± 0.03 ^a^	0.26 ± 0.03 ^a^	0.54 ± 0.07 ^b^	0.87 ± 0.15 ^c^

**Table 4:** Results of dietary fibre content in bread samples. Data are means ± SDs from three independent replicates. Means with different letters in rows indicate statistically significant differences between the bread samples (*p* < 0.05).

	Control	S30	S60	O30	O60	B30	B60	MG30	MG60
Dietary Fibre (%)									
Insoluble DF	2.16 ± 0.12 ^a^	3.10 ± 0.10 ^cd^	3.59 ± 0.13 ^f^	2.82 ± 0.08 ^bc^	3.28 ± 0.09 ^de^	2.88 ± 0.09 ^bc^	3.57 ± 0.24 ^ef^	2.77 ± 0.16 ^b^	3.53 ± 0.18 ^ef^
Soluble DF	0.62 ± 0.04 ^a^	1.14 ± 0.07 ^b^	1.37 ± 0.09 ^bc^	1.50 ± 0.10 ^cd^	1.82 ± 0.12 ^e^	1.29 ± 0.14 ^bc^	1.60 ± 0.17 ^de^	1.28 ± 0.07 ^bc^	1.59 ± 0.14 ^de^
Total DF	2.78 ± 0.14 ^a^	4.25 ± 0.13 ^b^	4.96 ± 0.17 ^c^	4.32 ± 0.14 ^b^	5.10 ± 0.05 ^c^	4.17 ± 0.13 ^b^	5.16 ± 0.10 ^c^	4.05 ± 0.15 ^b^	5.12 ± 0.16 ^c^

**Table 5:** Summarized results of specific loaf volume, water content, crust colour, texture properties, and sensory evaluation of bread samples. Data are means ± SDs from three independent replicates. Means with different letters in rows indicate statistically significant differences between the bread samples (*p* < 0.05). L*, a*, and b* represent the standard notations for color coordinates in the CIELAB color space, where L* indicates lightness, a* the red–green axis, and b* the yellow–blue axis.

	Control	S30	S60	O30	O60	B30	B60	MG30	MG60
**Specific volume (mL/g)**	3.42 ± 0.18 ^c^	2.86 ± 0.32 ^ab^	2.69 ± 0.23 ^a^	3.02 ± 0.22 ^ab^	2.63 ± 0.32 ^a^	3.22 ± 0.22 ^bc^	2.70 ± 0.19 ^a^	3.19 ± 0.08 ^bc^	2.84 ± 0.17 ^ab^
**Water content (%)**	45.54 ± 0.56 ^a^	45.99 ± 0.28 ^abc^	45.80 ± 0.64 ^ab^	46.91 ± 0.3 ^bcd^	47.46 ± 0.92 ^d^	47.42 ± 1.12 ^d^	49.06 ± 0.92 ^e^	46.33 ± 0.24 ^abcd^	47.21 ± 0.58 ^cd^
**Crust colour**									
L *	70.17 ± 1.36 ^e^	63.51 ± 1.45 ^de^	60.16 ± 1.56 ^bcd^	62.58 ± 3.18 ^cd^	55.85 ± 5.55 ^bc^	53.30 ± 4.72 ^ab^	48.03 ± 2.97 ^a^	61.27 ± 3.31 ^cd^	53.66 ± 3.06 ^ab^
a *	6.60 ± 1.00 ^a^	9.80 ± 1.06 ^b^	10.80 ± 0.46 ^bc^	9.75 ± 1.76 ^b^	11.24 ± 1.53 ^bc^	12.16 ± 1.2 ^bc^	12.91 ± 0.91 ^c^	10.01 ± 1.72 ^b^	12.34 ± 1.19 ^bc^
b *	32.90 ± 1.36 ^cd^	33.92 ± 0.66 ^d^	33.37 ± 0.76 ^cd^	33.57 ± 0.55 ^d^	31.55 ± 1.63 ^bcd^	29.77 ± 2.49 ^b^	26.96 ± 1.19 ^a^	33.09 ± 0.78 ^cd^	30.78 ± 1.38 ^bc^
**Hardness (N)**									
Day 0	4.55 ± 0.45 ^a^	6.98 ± 1.01 ^bc^	8.08 ± 0.78 ^c^	5.78 ± 0.82 ^ab^	7.74 ± 1.23 ^c^	4.72 ± 0.47 ^a^	5.52 ± 0.69 ^ab^	4.78 ± 0.86 ^a^	5.47 ± 0.82 ^ab^
Day 1	8.78 ± 0.69 ^abc^	10.47 ± 0.92 ^cd^	11.79 ± 1.56 ^d^	10.34 ± 1.29 ^bcd^	10.79 ± 1.40 ^cd^	7.11 ± 0.75 ^a^	9.26 ± 1.21 ^abc^	7.25 ± 0.96 ^a^	8.18 ± 1.62 ^ab^
Day 2	10.7 ± 0.79 ^ab^	12.05 ± 1.37 ^b^	15.14 ± 1.93 ^c^	12.80 ± 1.41 ^bc^	12.97 ± 1.48 ^bc^	9.44 ± 0.54 ^a^	11.92 ± 0.86 ^b^	11.17 ± 1.40 ^ab^	8.84 ± 0.94 ^a^
**Sensory evaluation**									
Shape and appearance	1.00 ± 0.00 ^a^	1.00 ± 0.00 ^a^	0.92 ± 0.20 ^a^	1.00 ± 0.00 ^a^	1.00 ± 0.00 ^a^	1.00 ± 0.00 ^a^	1.00 ± 0.00 ^a^	1.00 ± 0.00 ^a^	1.00 ± 0.00 ^a^
Crust properties	2.00 ± 0.00 ^a^	2.00 ± 0.00 ^a^	1.75 ± 0.27 ^a^	2.00 ± 0.00 ^a^	1.75 ± 0.27 ^a^	1.83 ± 0.26 ^a^	1.83 ± 0.26 ^a^	2.00 ± 0.00 ^a^	2.00 ± 0.00 ^a^
Crumb properties	3.92 ± 0.2 ^b^	3.83 ± 0.26 ^b^	3.50 ± 0.00 ^a^	4.00 ± 0.00 ^b^	3.50 ± 0.00 ^a^	4.00 ± 0.00 ^b^	3.83 ± 0.26 ^b^	4.00 ± 0.00 ^b^	3.92 ± 0.20 ^b^
Structure and elasticity	4.00 ± 0.00 ^b^	4.00 ± 0.00 ^b^	3.67 ± 0.26 ^a^	4.00 ± 0.00 ^b^	3.67 ± 0.26 ^a^	4.00 ± 0.00 ^b^	3.50 ± 0.00 ^a^	3.67 ± 0.26 ^a^	3.42 ± 0.20 ^a^
Aroma and taste	8.42 ± 0.20 ^a^	8.50 ± 0.00 ^a^	8.33 ± 0.26 ^a^	8.50 ± 0.45 ^a^	8.50 ± 0.32 ^a^	8.67 ± 0.26 ^a^	8.58 ± 0.20 ^a^	8.75 ± 0.27 ^a^	8.67 ± 0.26 ^a^
Sum of scores	19.33 ± 0.26 ^c^	19.33 ± 0.26 ^c^	18.17 ± 0.41 ^a^	19.50 ± 0.45 ^c^	18.42 ± 0.49 ^ab^	19.50 ± 0.45 ^c^	18.75 ± 0.27 ^abc^	19.42 ± 0.49 ^c^	19.00 ± 0.55 ^bc^

## Data Availability

The data presented in this study are available on request from the corresponding author. The dataset forms part of an ongoing doctoral research project and will be deposited in the institutional repository of the Biotechnical Faculty, University of Ljubljana upon completion of the doctoral thesis.

## Supplementary Materials

The following supporting information can be downloaded at https://www.mdpi.com/article/10.3390/foods15061029/s1. Table S1. Characteristics of the phenolic compounds in buckwheat extracts as analyzed by LC-MS/MS; Table S2. Concentrations of phenolic compounds and benzoxazinoids (µg/g DW) in the free fraction of dough samples prior to baking; Table S3. Concentrations of phenolic compounds and benzoxazinoids (µg/g DW) in the bound fraction of dough samples prior to baking; Figure S1. Relative increase in bread hardness in 48 h period; Figure S2. Specific reactivities of selected compounds toward FC reagent; Figure S3. Specific reactivities of selected compounds toward DPPH reagent.

## Abbreviations

The following abbreviations are used in this manuscript:

LC-MS/MS	Liquid Chromatography–Tandem Mass Spectrometry
BOA	2-Benzoxazolinone
MBOA	6-Methoxy-2-benzoxazolinone
AVN A	Avenanthramide A
AVN B	Avenanthramide B
AVN C	Avenanthramide C
S30/S60	Bread formulations prepared with 30% and 60% germinated spelt
O30/S60	Bread formulations prepared with 30% and 60% germinated oat
B30/B60	Bread formulations prepared with 30% and 60% germinated buckwheat
MG30/MG60	Bread formulations prepared with 30% and 60% mixed germinated grains
TPC	Total Phenolic Content
AA	Antioxidant Activity
FC	Folin–Ciocalteu
DPPH	2,2-Diphenyl-1-picrylhydrazyl
SDF	Soluble Dietary Fibre
ISF	Insoluble Dietary Fibre
TDF	Total Dietary Fibre
