# Supplementary material for: Multigrain Bread: Impact of Germinated Grain Supplement on Phytochemical Profile and Technological and Nutritional Properties
Source: Foods. 2026 Mar 16;15(6):1029. doi: 10.3390/foods15061029 (PMC13025957; doi:10.3390/foods15061029)
Supplement: Supplementary file 1 [file foods-15-01029-s001.zip › foods-4169908-supplementary.pdf]

Supplementary Table S1. Characteristics of the secondary metabolites in determined in bread extracts as analysed by LC-MS/MS

	Rt (min)	ESI-Precursor ion	Fragment ion	Standard LOQ ng/column	LOD ng/column	Sample LOQ ng/column	LOD ng/column	Recovery (%)	Linear regression equation	R^2
Catechin	3.36	289.1	139.1	0.45	0.13	0.83	0.31	94.33	246259x	0.9952
trans-p-Coumaric acid	7.51	162.90	119.47	0.53	0.16	0.78	0.27	93.77	36029x	0.9998
Isoorientin	7.88	447.2	327.2	0.79	0.24	1.23	0.51	96.2	322147x	0.9905
Schaftoside	8.01	563.16	353.06	0.27	0.08	0.48	0.17	95.66	243584x	0.9973
Orientin	8.11	447.2	327.2	0.25	0.08	0.52	0.18	93.12	128664x	0.9923
BOA	8.13	133.93	90.93	0.09	0.03	0.21	0.07	94.28	76376x	0.9727
trans-ferulic acid	8.47	192.95	133.94	0.11	0.04	0.19	0.06	96.78	24041x	0.9996
Vitexin	8.73	431.2	311.2	0.12	0.04	0.15	0.07	93.47	314287x	0.9889
Rutin	8.69	609.2	301.2	0.08	0.03	0.18	0.07	95.31	185008x	0.9783
MBOA	9.26	163.95	148.92	0.17	0.05	0.39	0.14	95.34	34983x	0.982
AVN-C	10.21	314.24	270.26	0.15	0.05	0.19	0.07	93.11	292435x	0.9945
AVN-A	11.22	298.11	254.09	0.16	0.06	0.25	0.08	91.24	491722x	0.9899
AVN-B	11.58	328.09	284.23	0.11	0.04	0.15	0.05	90.78	380217x	0.9977

Supplementary Table S2. Concentrations of phenolic compounds and benzoxazinoids (μg/g DW) in the free fraction of dough samples prior to baking. Data are means ± SD from three independent replicates. Means with different letters in rows indicate statistically significant differences between the dough samples ($p < 0.05$). ND—compound not detected.

Phenolic compound	Content of benzoxazinoids and phenolics (μg/g DW) in samples of dough								
	**Control**	**S30**	**S60**	**O30**	**O60**	**B30**	**B60**	**MG30**	**MG60**
**Free fraction**									
AVN A	ND	ND	ND	7.97^{c} ± 0.17	12.81 ± 0.04^{d}	ND	ND	2.84 ± 0.14^{a}	5.11 ± 0.21^{b}
AVN B	ND	ND	ND	10.97 ± 0.26^{c}	18.22 ± 0.13^{d}	ND	ND	3.7 ± 0.25^{a}	6.41 ± 0.72^{b}
AVN C	ND	ND	ND	9.54 ± 0.6^{c}	17.64 ± 0.03^{d}	ND	ND	3.53 ± 0.10^{a}	6.72 ± 0.33^{b}
BOA	ND	7.03 ± 0.31^{c}	12.81 ± 0.07^{d}	ND	ND	ND	ND	2.69 ± 0.45^{a}	3.90 ± 0.26^{b}
MBOA	ND	1.78 ± 0.08^{c}	3.35 ± 0,21^{d}	ND	ND	ND	ND	1.00 ± 0.08^{a}	1.45 ± 0.06^{b}
Orientin	ND	ND	ND	ND	ND	13.01 ± 0.88^{c}	21.07 ± 0.14^{d}	3.9 ± 0.04^{a}	6.88 ± 0.83^{b}
Isoorientin	ND	ND	ND	ND	ND	14.46 ± 1.81^{c}	25.06 ± 1.20^{d}	4.28 ± 0.45^{a}	9.13 ± 0.03^{b}
Rutin	ND	ND	ND	ND	ND	19.93 ± 1.36^{c}	33.1 ± 2.52^{d}	6.91 ± 0.22^{a}	12.88 ± 1.35^{b}
Vitexin	ND	ND	ND	0.28 ± 0.01^{a}	0.54 ± 0.05^{b}	28.91 ± 1.26^{e}	41.8 ± 2.38^{f}	9.72 ± 0.18^{c}	17.72 ± 1.48^{d}
Catechin	ND	ND	ND	ND	ND	19.28 ± 0.81^{c}	32.02 ± 1.85^{d}	5.54 ± 0.54^{a}	9.06 ± 0.40^{b}
Epicatechin	ND	ND	ND	ND	ND	10.8 ± 0.65^{c}	17.46 ± 0.92^{d}	3.5 ± 0.25^{a}	7.04 ± 0.22^{b}
Schaftoside	3.85 ± 0.21^{bc}	5.51 ± 0.41^{e}	6.82 ± 0.34^{f}	3.52 ± 0.19^{b}	3.13 ± 0.05^{ab}	3.26 ± 0.11^{ab}	2.95 ± 0.34^{a}	4.53 ± 0.89^{cd}	4.71 ± 0.08^{d}
Schaftoside isomer	3.28 ± 0.25^{bc}	4.28 ± 0.22^{d}	5.28 ± 0.16^{e}	3.04 ± 0.1^{ab}	2.86 ± 0.04^{ab}	2.98 ± 0.17^{ab}	2.38 ± 0.1^{a}	3.43 ± 0.17^{c}	3.73 ± 0.19^{cd}
trans-ferulic acid	0.27 ± 0.03^{a}	0.42 ± 0.02^{c}	0.60 ± 0.02^{d}	0.63 ± 0.05^{d}	0.76 ± 0.08^{e}	0.25 ± 0.05^{a}	0.25 ± 0.04^{a}	0.31 ± 0.01^{ab}	0.33 ± 0.04^{ab}
cis-ferulic acid	0.17 ± 0.03^{ab}	0.25 ± 0.01^{c}	0.32 ± 0.08^{d}	0.14 ± 0.01^{a}	0.23 ± 0.02^{bc}	0.23 ± 0.03^{bc}	0.13 ± 0.03^{a}	0.20 ± 0.01^{b}	0.48 ± 0.01^{e}
trans-*p*-Coumaric acid	0.23 ± 0.01^{a}	0.61 ± 0.05^{b}	0.80 ± 0.08^{c}	0.68 ± 0.02^{bc}	0.86 ± 0.05^{c}	1.23 ± 0.01^{e}	2.25 ± 0.12^{f}	0.77 ± 0.06^{c}	1.06 ± 0.07^{d}

Supplementary Table S3. Concentrations of phenolic compounds and benzoxazinoids (μg/g DW) in the bound fraction of dough samples prior to baking. Data are means ± SD from three independent replicates. Means with different letters in rows indicate statistically significant differences between the dough samples ($p < 0.05$). ND—compound not detected.

Phenolic compound	Content of benzoxazinoids and phenolics (μg/g DW) in samples of dough								
	**Control**	**S30**	**S60**	**O30**	**O60**	**B30**	**B60**	**MG30**	**MG60**
**Bound fraction**									
Orientin	ND	ND	ND	ND	ND	0.62 ± 0.14^{b}	0.73 ± 0.02^{c}	0.38 ± 0.06^{a}	0.62 ± 0.01^{b}
Isoorientin	ND	ND	ND	ND	ND	0.49 ± 0.03^{b}	0.48 ± 0.05^{b}	0.28 ± 0.01^{a}	0.51 ± 0.06^{b}
Vitexin	ND	ND	ND	ND	ND	0.9 ± 0.04^{c}	1.52 ± 0.04^{d}	0.25 ± 0.03^{a}	0.53 ± 0.05^{b}
Schaftoside	0.31 ± 0.03^{b}	0.73 ± 0.18^{de}	1.53 ± 0.48^{f}	0.34 ± 0.05^{b}	0.24 ± 0.03^{a}	0.32 ± 0.06^{b}	0.23 ± 0.03^{a}	0.49 ± 0.02^{c}	0.62 ± 0.06^{d}
Schaftoside isomer	0.17 ± 0.03^{ab}	0.41 ± 0.01^{e}	0.63 ± 0.15^{f}	0.2 ± 0.01^{b}	0.15 ± 0.01^{a}	0.21 ± 0.02^{b}	0.19 ± 0.06^{ab}	0.25 ± 0.03^{bc}	0.34 ± 0.04^{d}
trans-ferulic acid	58.81 ± 2.99^{c}	133.33 ± 18.28^{f}	168.73 ± 16.31^{g}	83.2 ± 13.61^{d}	107.27 ± 9.96^{e}	50.5 ± 1.65^{b}	43.79 ± 1.24^{a}	87.46 ± 0.40^{d}	132.12 ± 13.59^{f}
cis-ferulic acid	15.87 ± 2.38^{bc}	28.18 ± 0.52^{e}	37.18 ± 3.58^{f}	24.54 ± 0.59^{d}	30.41 ± 3.48^{ef}	12.45 ± 0.81^{b}	8.80 ± 0.83^{a}	23.27 ± 2.71^{d}	27.08 ± 0.94^{e}
trans-*p*-Coumaric acid	0.73 ± 0.08^{a}	3.18 ± 0.36^{b}	5.34 ± 0.33^{d}	4.02 ± 0.34^{c}	6.79 ± 0.4^{e}	1.14 ± 0.14^{a}	1.39 ± 0.15^{a}	2.61 ± 0.18^{b}	4.46 ± 0.14^{c}
cis-*p*-Coumaric acid	0.17 ± 0.03^{a}	0.47 ± 0.03^{b}	0.58 ± 0.03^{b}	0.7 ± 0.07^{c}	1.1 ± 0.12^{d}	0.15 ± 0.01^{a}	0.16 ± 0.02^{a}	0.47 ± 0.06^{b}	0.62 ± 0.12^{bc}

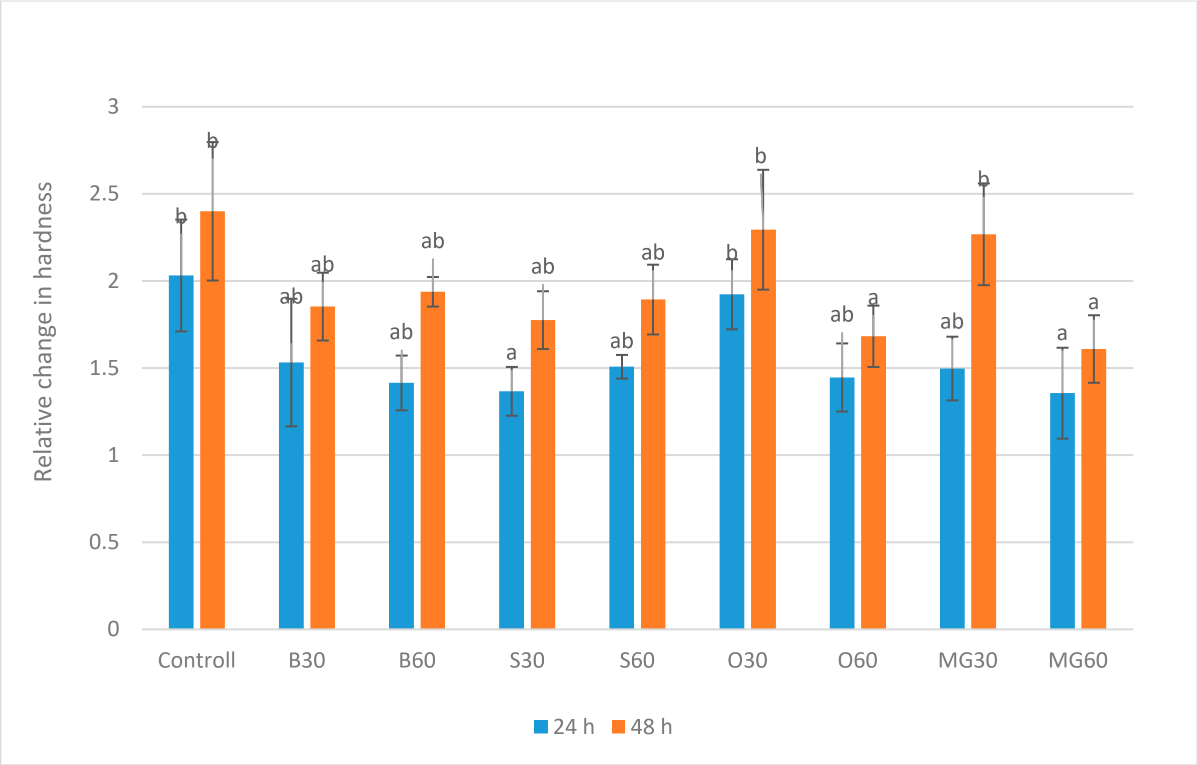

Supplementary Figure S1. Relative increase in bread hardness in 24 h and 48 h period. Means with different letters indicate statistically significant differences between the different bread formulations at same time after baking ($p < 0.05$)

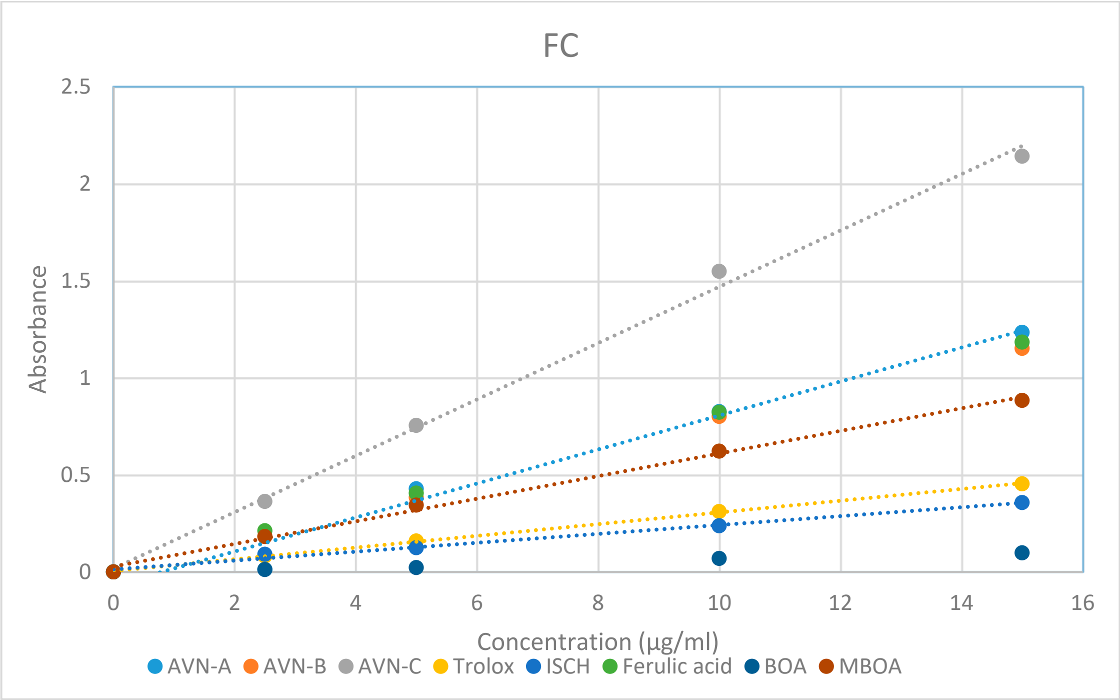

Supplementary Figure S2. Specific reactivities of selected compounds toward FC reagent

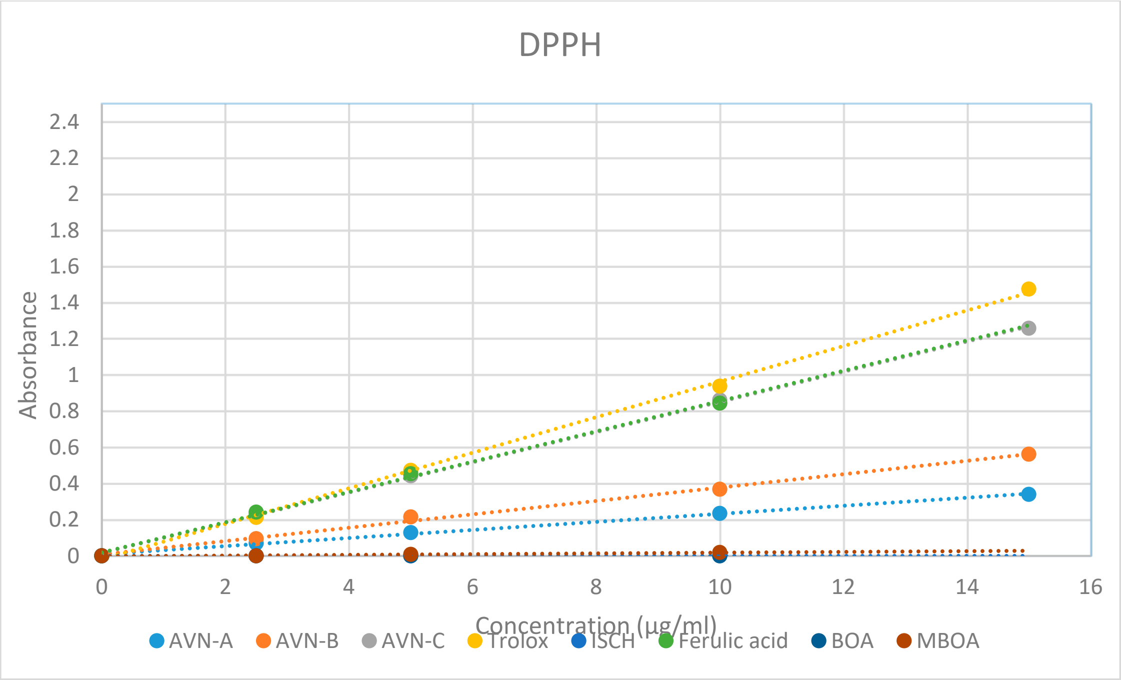

Supplementary Figure S3. Specific reactivities of selected compounds toward DPPH reagent
